# Functional Principles of Morphological and Anatomical Structures in Pinecones

**DOI:** 10.3390/plants9101343

**Published:** 2020-10-12

**Authors:** Haejin Bae, Jinhee Kim

**Affiliations:** Biomimicry Team, Division of Ecological Information, National Institute of Ecology, Seocheon-gun 33657, Korea; hjbae@nie.re.kr

**Keywords:** biomimicry, cross-section structure, hygroscopy, morphological movement, pinecone

## Abstract

In order to better understand the functions of plants, it is important to analyze the internal structure of plants with a complex structure, as well as to efficiently monitor the morphology of plants altered by their external environment. This anatomical study investigated structural characteristics of pinecones to provide detailed descriptions of morphological specifications of complex cone scales. We analyzed cross-sectional image data and internal movement patterns in the opening and closing motions of pinecones, which change according to the moisture content of its external environment. It is possible to propose a scientific system for the deformation of complex pinecone for the variable structures due to changes in relative humidity, as well as the application of technology. This study provided a functional principle for a multidisciplinary approach by exploring the morphological properties and anatomical structures of pinecones. Therefore, the results suggest a potential application for use in energy-efficient materials by incorporating hygroscopic principles into engineering technology and also providing basic data for biomimicry research.

## 1. Introduction

Because plant traits are influenced by environmental changes and signals [1], it is important to obtain information about plant characteristics by examining their internal mechanisms to understand structural changes. Plant structures can create complex movements through unique morphological changes and these movements are closely related to the complex structure of each plant species [2,3,4]. For instance, the movement of the *mimosa pudica* leaf, a representative plant that exhibits visible movement by external stimulation, is activated by osmotic phenomena, ion substitution, and enzymes via a special internal structure [5,6].

When considering the unique characteristics of plants, principle analysis and original technology development in biomimicry research and plant fields are conducted. Biomimicry is a concept derived from commercialization and is a convergence process that mimics the structure, function, mechanism, and ecosystem of living organisms that have adapted to the environment through evolution [7]. In other words, it is an interdisciplinary field that would solve the environmental and social problems by applying the principles and characteristics of nature including biological and ecological resources. Depending on changes in the external environment, as a plant changes, one can diagnose either an entire structure or a part of plant growth, stress, or movement. Moisture is an important parameter both internally and externally, as it plays an important role in forming a main component in plant structure.

Pinecones have received research attention due to their flexible nature of outer scale movement. When humidity is high, the fiber tissue outside the scale contracts, whereas when humidity is low (dry), the fibers of the tissue inside the scale expand [8], causing the scale to open and close. The changes in cone mechanical behavior is speculated to form the structure and properties of fibers. Outer fibers of cone scales could be used to control the mechanical morphology for pinecones. Thus, pinecones in high relative humidity are hampered and bent outward. The hierarchical structure of porous types with different sizes inside the scale leads to the opening and closing of the scale in relation to water movement and evaporation [9]. The specific mechanisms of a pinecone opening and closing is related to survival strategy, which allows the pine to disperse seeds over longer distances [10]. Consequently, the shape of the pinecone changes because the pinecone relates to water (moisture) in its external environment [11,12]. Since the pinecone is composed of dead cells, the operation of implementing the hygroscopic mechanism of the pineal scales can be disseminated into a passive mechanism [13]. In addition, even though the pinecone is composed of dead cells [14], it is not infected with fungus because the tannin component in the pinecone inhibits the growth of fungi [15].

Although the hydromorphic movements of pinecones were geometrically and dynamically evaluated [16,17], the distinct characteristics involved in the biological and ecological characteristics have not yet been explored. Therefore, it is worth defining the structural and morphological studies of pinecones from a biological and ecological point of view. The aim of this study was to analyze the anatomical and morphological features of internal and external pinecones using different forms of microscopes in order to provide comprehensive structural characteristics. To derive the possible ways of delivering new technology from hygroscopic functions of pinecones, we measured the opening and closing angles of cone scales using microcomputed tomography. In addition, we investigated the distribution and size of pores on the surface of cone scales that could affect moisture absorption and dehumidification in pinecones in order to indirectly determine water absorption ability.

## 2. Results and Discussions

### 2.1. Estimation of Angle Observation from 3D Images

**Figure 1:**
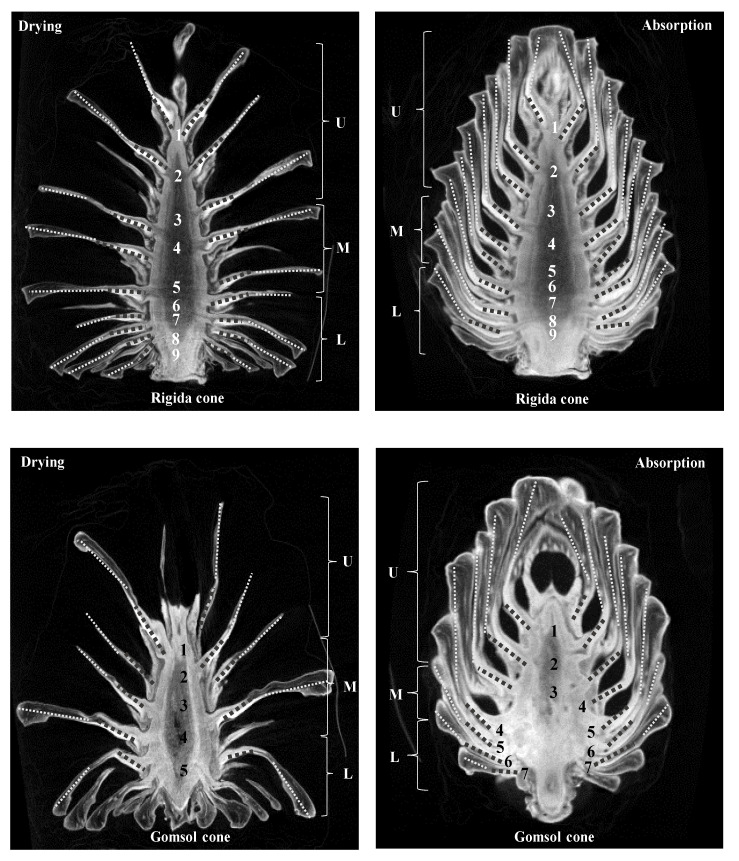
Numbers and locations for measuring opening and closing angles of “rigida” and “gomsol” cones according to drying and water absorption. Bract (•••••), Scale (·····).

The change in pinecone shape is related to the amount of water that subsists among external environmental factors. The pinecone is alive when it fruits on the tree, whereas the cone comprises dead cells when it falls off [18]. Thus, the operation of implementing the hygroscopic mechanism of pinecone scales can be categorized as a passive mechanism. Since there is a tissue that holds cone scales together at the base, pinecone composing of dead cells maintains the shapes. The scale angles were theoretically quantified using the program to compare opening (unfolding) and closing (folding) differences. Analyzing the opening and closing angles of the cone scales provided minimal information for measuring the plant structural parameters of the cones. In this study, the cone scale was classified into two parts: bracts and scales. Dawson et al. described in detail that the scale is composed of a bract scale and ovuliferous scale including fibers and sclerids [19]. The angle of movement of the bracts (bold dots) and the scales (dots) supporting the cones were measured (Figure 1).

When the surrounding environment is dry, cones show an angle at which the specimen opens. When the humidity of the surrounding environment is high, the specimen closes. 

With “rigida” (Table 1), the angle of opening the scale in the dry state increases toward the lower part. The opening angles during drying descends in the order from upper (no. 1) to middle (no. 5) and then to lower (no. 9). The opening angles of the left bracts were 42.6°, 86.7°, and 133.1°, and the opening angles of the right bracts were 45.3°, 69.0°, and 115.5°. The opening angles of the left scales were 23.6°, 89.6°, and 111.6°, and the opening angles of the right scales were 45.5°, 86.0°, and 120.7°. The opening angles were similar for both the left and right, and the difference between the left and right was due to the differences in shape and length of the cone. When the pinecone was hygroscopically observed, the closing angle of the scale got larger when moving from the upper part toward the lower part. The closing angle of the thread in the upper, middle, and lower areas creates a conical shape as the cone closes. Thus, the actual length of the lower part of the cone seems to move greatly. 

The closing angles of the “rigida” upon absorption descends in order from upper (no. 1) to middle (no. 5) and then to lower (no. 9). The closing angles for the left bracts (bold dots) were 30.0°, 45.6°, and 60.0°. The closing angles of the right bracts were 30.5°, 43.1°, and 83.4°. The closing angles of the left scales were 12.4°, 2.7°, and 25.6°, and the closing angles of the right were 16.7°, 4.8°, and 28.9°. This results show that when cones close, the bract portions moves considerably more than the scales, perhaps explaining why multiscale structures of fibers inside cone scales lead to hygro-elastic properties [20].

Table 1 presents findings regarding the opening and closing angles of the relatively short and round-shaped “gomsol” (Table 1). In the hygroscopic state of the “gomsol”, the closing angle of the scales increased and it became larger moving toward the lower part rather than the upper (no. 7). In the closing angle of the scales, the closing angles of the lower scales were larger than in the upper and middle sections. The irregular closing angle of the upper left scale is believed to be due to the internal shape. The closing angles on the right bracts were 23.9°, 57.1°, and 56.9° from upper to lower. The closing angle of the right upper scale was also larger at the lower than at the upper and middle. It was confirmed that the “gomsol” was completely closed in appearance as the moisture was absorbed, but a gap between the central axis and the space was observed. Baley (2002) explained that the phenomenon of scale opening and closing is due to the axis of the fiber and the direction of the fiber [21].

In the dry state, the opening angle of the scales got larger moving toward the lower part from the upper. The opening angle of the scales during drying descended in order from upper (no. 1) to middle (no. 3) and then to lower (no. 5). The opening angles of the left bracts were 12.6°, 56.5°, and 69.4°, and the opening angles of the right bracts were 26.5°, 52.3°, and 77.2°. The opening angles of the left scales were 4.4°, 49.1°, and 84.8°, and the opening angles of the right scale were 4.8°, 53.4°, and 136.7°. The reason the opening angle of the lower scale sharply increases is that the bracts and the upper scales open downward, so a sharp difference in angle is presented.

The phenomena are supported by the fact that cones absorb water first and then quickly transfer moisture from the lower to upper scales. It is hypothesized that the relative difference between the hard wood in the upper part and the soft wood in the lower part caused the moisture absorption to be different. Therefore, studies related to the folding motion of pinecones can influence biomimicry technology that expresses various movements at a low cost [22]. Further, the opening and closing mechanism may provide insight into novel devices, such as a passive actuated humidifier, or into architectural applications.

### 2.2. Thickness Changes of Internal Structure

**Figure 2:**
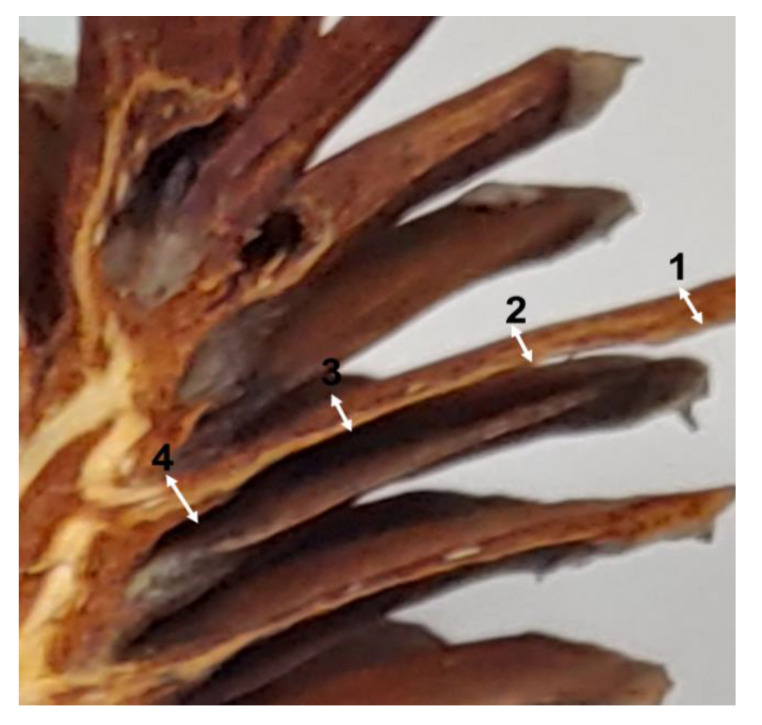
Positions of cone scale to measure the change in thickness.

**Figure 3:**
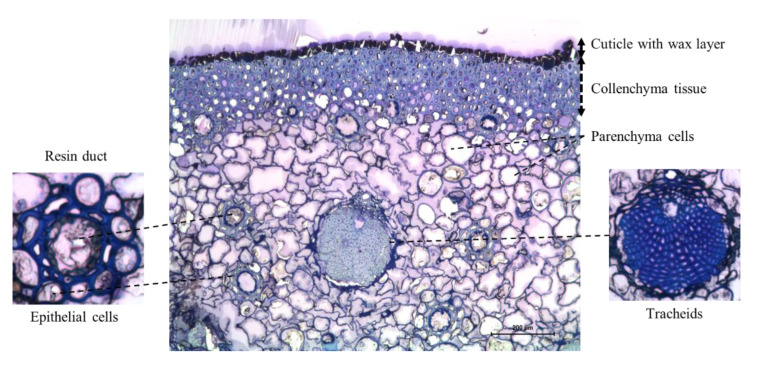
Cone scale of cross-section view in optical microscope.

When the “rigida” was in a dry and moisture-absorbing state, the scales were cut to measure using a stereomicroscope. The thickness change was measured by setting the position of 1 to 4 locations from the outside of the scales to the central axis of each cones (Figure 2). In the hygroscopic state, the *parenchyma* tissues of scales were more swollen than in the dry state (Figure 3). Depending on the moisture absorption state, the change in the thickness of the scale was different by position of the 1–4 scale. When dried scales were completely absorbed, the percentage of the changes in thickness was about 1.7–34.0% for “rigida”, and about 4.7–32.7% for “gomsol” (Table 2). The difference between the opening and closing angles of the two pinecones is due to the outer and inner shapes and length of the cone scales as shown in Figure 1. In other words, the cone’s opening and closing angles do not depend on the species of pine but on the shapes of the pinecones. Since the movements of the cones are controlled by the amount of water in the bracts and scales, the cone parts with bracts was thicker and absorbed more water at the bottom part (position 3 and 4) than at the upper part (position 1 and 2).

### 2.3. Cross-Section Structure of Cone Scale

To determine the internal structure of the pine scales and to analyze the specificity of the intracellular structure related to the hygroscopic function, the inside of the scale was observed with an optical microscope (Figure 3). The sub-epidermis is composed of a cuticle with a wax layer and *collenchyma* tissues. The *tracheids* have a thick secondary wall and the inner cells are structured with narrow and long tubular cells. The *tracheids* plays a role in maintaining the pinecone structure. Resin ducts are commonly known to exist in the cortex and are generally surrounded by *epithelium* [23,24]. Several resin ducts of the pinecone are found in the secondary tissue. The resin duct is comprised of *epithelial* cells surrounding lumen that consist of sheath cells. In most *Pinus* species, the opening and closing of pinecone scales is caused by water movement and evaporation into the tissue, which occurs due to the expansion and loosening of the cell wall or internal structure, so only the opening and closing movement is visible externally. The scale comprises of perforated tubular resin ducts and various-sized fibrous tissues. Increasing the pore surface area and volume of the scale improves the hygroscopic function. *Parenchyma* consist of living cells and are widely distributed throughout the tissues. The cells typically show a thin cell wall, but could not be differentiated because the structures were not specialized. In the case of pinecones, the size and shape of *parenchyma* cells changed depending on water content. This was because when cones absorb water, cells become a form capable of holding a certain amount of water.

### 2.4. Internal and Surface Structure of Cone Scale

**Figure 4:**
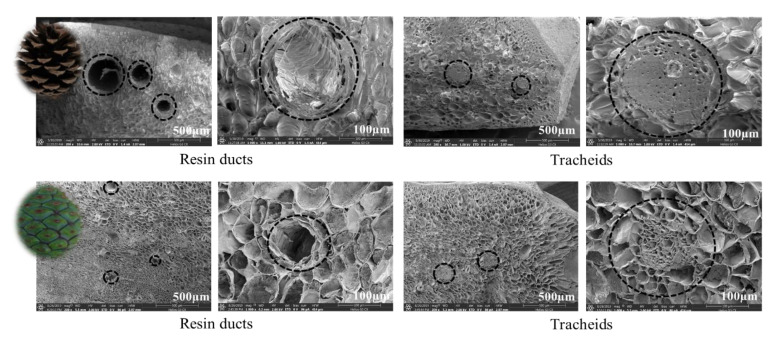
FE-SEM (Field Emission Scanning Electron Microscope) images of internal structure of mature and immature cones.

**Figure 5:**
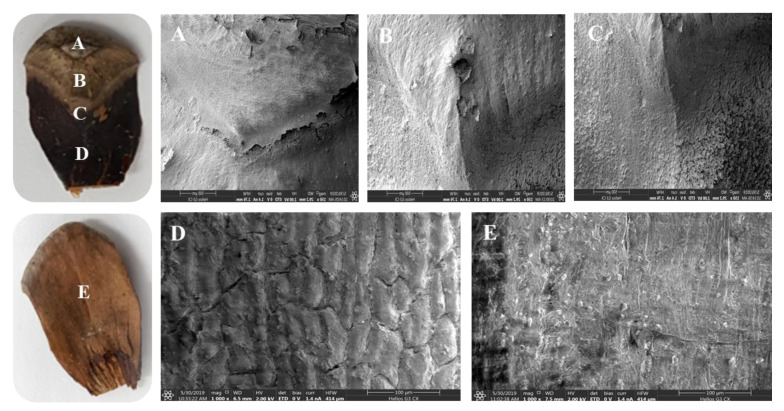
FE-SEM images of surface structure of dorsal (**A**–**D**) and ventral (**E**) cones.

To identify the structural specificity (density, morphology, etc.) of the cone scale, internal and surface structure images were observed using FE-SEM (Figure 4). In mature scales, the major tissues found in the surface of the *parenchyma* were pierced tubular resin ducts. Different sizes of *tracheids* with a thick wall structure were found inside mature scales. As a result of observing the surface of immature cones to compare the structural differences to mature cones, a large number of resin ducts were also found in the surface structure of immature scales. When comparing the structure of *tracheids* between mature and immature scales, coarser structures were found in immature surfaces than in the dense *tracheids* of the mature surface. In addition, dorsal (A~D) and ventral (E) surfaces of cone scales showed different surface structures (Figure 5). Various species have different surface structures because silver fir cones showed different structures in comparison to *Pinus* cones [25]. The *parenchyma* in dorsal and ventral surfaces of cone scales are surrounded by fibrous tissue. The ventral part of the scale has a softer texture than the dorsal part because the ventral tissue has a relatively small amount of *tracheids*. When comparing the *tracheids* structure, the surface of the mature scales showed dense structures, whereas the immature scales revealed coarse structures.

### 2.5. Pore Distribution of Cone Scales

**Figure 6:**
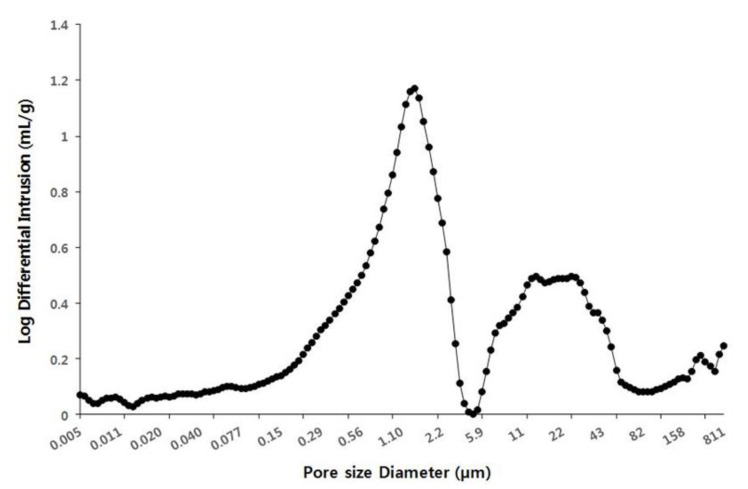
Porosity and density distribution on the cone scale surface.

The pore size and porosity of the scales were measured to understand the relationship between moisture-absorption function and pore distribution of cone scales (Figure 6). More than 145 pores were present in 2–3 scales and the pore size ranged from 0.005 µm or less to 810 µm or more. It showed that the pore size of 1 µm was the most distributed on the surface of cone scales. This result determined that the increased pore surface area and increased pore volume improved the moisture absorption function of cone scales. However, the image of the pore distribution on the surface of the cone scale was not shown in this study. Therefore, the relationship between the pore distribution and hygroscopic power could be more clearly revealed by measuring the pore distribution of different types of pinecones in the future.

## 3. Materials and Methods

### 3.1. Plant Material

Mature and immature pinecones (i.e., “rigida” (*Pinus rigida*) and “gomsol” (*Pinus thunbergii*)) were collected from Jane Goodall road at the National Institute of Ecology. First, we prepared five cones from the same species. After the cones were washed for 6 min in 20% ethanol, they were dried at room temperature and used as experimental materials. In this study, pinecone samples were allowed to absorb water and dry naturally at room temperature, which replicates what happens in their external environment. Three cone scales from each cone were selected and analyzed for all experiments. They were performed in triplicate and repeated three times.

### 3.2. Microcomputed Tomography Analysis

The dried pinecones were measured and once the cones were closed (water absorbed), they were measured while wrapped in a plastic wrap to prevent drying during shooting. The cones were photographed using SkyScan high-resolution, microcomputed tomography (SkyScan 1076, Kontich, Belgium) to measure the change in the internal scales due to the humidity of cones. The Hounsfield unit (HU) and tissue mineral density calibration procedures were performed with CTAn software (CT-Analyzer, v.1.6.1, SkyScan, Kontich, Belgium). The mean grayscale value was used to calibrate the micro-CT images with HUs, as well as to generate a calibration curve between the grayscale color in each pixel and the corresponding mineral density in gHA/cm^3^. The threshold value was determined by analyzing the images using an edge-detection algorithm (ImageJ v1.37). The tissue mineral density threshold obtained by this edge-detection procedure was 0.40 gHA/cm^3^. To measure the angles according to the opening (unfolding) and closing (folding) of the cone, 3D visualization and analysis of the micro-CT images were performed using NRecon, CTvox, and CTAn (Skyscan, Bruker, Kontich, Belgium) software, respectively. Based on the photographed data, the vertical line of the central axis of the cross-section of each cone was determined, and the angle was measured using left and right symmetry. To compare the angular differences between parts, the commonly called scale is divided into bract and scale. The angles of opening and closing of the cone scales were measured after analyzing the three-dimensional (3D) CT images of the structural changes of the scales. Further, the angle values were measured from the central axis. 

### 3.3. Dissecting-Microscope Appearance

In our analysis, we used two raw pinecones from the “rigida” (*Pinus rigida*) and “gomsol” (*Pinus thunbergii*) families, species that are commonly found in South Korea. Each type of pinecone was washed with an ultrasonic cleaner containing 20% ethanol and then dried completely at room temperature. The cones were cut in half to measure the thickness change of scales in the conditions of a moisture-absorbing and dry state. The thickness changes inside the scale were photographed using a stereomicroscope (Leica M205C, Leica Microsystems, Wetzlar, Germany) with a resolution of 500 µm. Then, the thickness length was measured using Image J.

### 3.4. Optical Microscope Analysis

The cone scales were cut (1 mm × 1 mm) to analyze the internal structure. Samples were fixed with glutaraldehyde (1%, *v/v*) and paraformaldehyde (2%, *v/v*) in 50 mM CAB (sodium cacodylate buffer, pH 7.2) at room temperature for 4 h. After washing with CAB three times for 15 min, the samples were dehydrated to a graded ethanol series (30%, 50%, 70%, 80%, 90%, and 95%) for 15 min each and then dehydrated through 100% ethanol three times for 20 min each. They were then infiltrated in mixtures of 100% ethanol to LR white resin (1:1) for 4 h and vacuumed for 30 min. After neutralizing the pure LR white resin and the new LR white resin for 24 h at 60 ° C, the thin section (1 µm) was fixed on the grid and colored with toluidine blue-O for 2 min. The internal structure of the scales was observed under an optical microscope (ZEISS, Oberkochen, Germany).

### 3.5. FE-SEM (Field Emission Scanning Electron Microscope) Analysis

The sample was placed in an Eppendorf tube with 1 mL of fixative (2% glutaraldehyde and 2% paraformaldehyde in 0.05 M cacodylate buffer pH 7.2), vacuumed for 30 min, and fixed at room temperature for 4 h. The fixative was removed and washed by changing three times with 0.05 M cacodylate buffer every 20 min. After fixing for 1 h with 1% OsO_4_ at room temperature, the sample was washed with 0.05 M cacodylate buffer three times at 20 min intervals. The sample was dehydrated with 30%, 50%, 70%, 90%, and 95% ethanol stepwise at 20 min intervals, and then 100% ethanol was used for dehydration stepwise at 20 min intervals. After the critical point of drying, the surface structure of the cone scale was observed using a field emission scanning electron microscope (Helios G3 CX, FEI Czech Republic Ltd., Brno, Czech Republic) under the accelerating voltage 1 to 3 kv after PT coating.

### 3.6. Mercury Intrusion Porosimetry Analysis

In the mercury intrusion measurement, the pore size and surface area of the sample were obtained using mercury under high pressure in a vacuum overnight to remove the residual moisture in the pinecone. The porosity measurement relies on the capillary phenomenon as the liquid penetrates into the fine pores. Penetration takes place only when pressure is applied from the outside, and the smaller the pore size, the higher the pressure obtained. Using the property of mercury with strong cohesion, pressure was applied from the outside by setting 0.2 psia to 33,000 psia to equilibrate for 10 s. The volume value of mercury was measured by using a mercury intrusion meter (Hg porosimeter, AutoPore IV Series, Norcross, GA, USA). 

## 4. Conclusions

Understanding biological entities are a fundamental approach to find new solution techniques. Because the morphological changes of pinecones can be applied to technology development, it was worthwhile to study the opening and closing mechanisms, as well as the structural characteristics that respond to hygroscopic action. In addition, a unique structure and system that biomimic the motion of a pinecone essentially can be applied to a hygromorphic model [26]. The results of this study can be used as scientific data for future biological and ecological characteristic analyses and applications based on the characteristic principles of a plant (pinecones) with a moisture absorption function. Furthermore, this study provides insights that can be applied to eco-friendly and energy-efficient materials by combining the principle of hygroscopic function with engineering technology.

## Figures and Tables

**Table 1:** Opening and closing angles of cone scales according to drying and water absorption.

**Rigida**	**Drying Angle (** **°)**				**Absorption Angle (** **°)**			
**Scale no.**	**Left**		**Right**		**Left**		**Right**	
	Bract	Scale	Bract	Scale	Bract	Scale	Bract	Scale
1	42.6	23.6	45.3	45.5	30.0	12.4	30.5	16.7
2	58.6	55.4	46.7	47.3	33.3	5.1	36.6	7.4
3	68.6	78.8	65.9	63.2	43.8	3.0	48.3	3.8
4	77.8	82.3	74.6	77.6	50.4	1.7	52.5	1.5
5	86.7	89.6	69.0	86.0	45.6	2.7	43.1	4.8
6	87.5	102.6	80.0	88.3	46.2	7.7	54.1	5.3
7	96.8	119.1	90.3	113.5	59.5	9.1	63.0	8.9
8	103.5	112.2	97.9	117.4	75.6	18.9	80.5	19.0
9	133.1	111.6	115.5	120.7	60.0	25.6	83.4	28.9
**Gomsol**	**Drying Angle (** **°)**				**Absorption Angle (** **°)**			
**Scale no.**	**Left**		**Right**		**Left**		**Right**	
	Bract	Scale	Bract	Scale	Bract	Scale	Bract	Scale
1	18.63	39.8	23.9	21.7	12.6	4.4	26.5	4.8
2	7.2	45.5	28.1	12.9	41.6	54.8	48.2	29.6
3	3.3	56.1	45.5	6.5	56.5	49.1	52.3	53.4
4	4.4	48.8	57.1	2.3	60.4	56.3	67.6	83.2
5	15.5	47.2	41.5	9.61	69.4	84.8	77.2	136.7
6	43.5	65.6	50.3	21.8	-	-	-	-
7	70.3	95.7	56.9	44.5	-	-	-	-

**Table 2:** Changes in thickness of the cone scale based on dryness and absorption status.

Position	Rigida			Gomsol		
	Dryness (µm)	Absorption (µm)	Change (%)	Dryness (µm)	Absorption (µm)	Change (%)
1	367.9 ± 35.4	374.0 ± 24.5	1.7	325.3 ± 96.4	384.3 ± 19.3	18.2
2	264.8 ± 40.9	287.9 ± 46.3	8.7	168.1 ± 79.3	176.1 ± 107.2	4.7
3	199.5 ± 36.4	231.7 ± 54.0	16.1	101.5 ± 44.0	132.3 ± 46.5	30.3
4	117.4 ± 22.1	157.4 ± 53.3	34.0	112.3 ± 9.3	149.1 ± 35.7	32.7
